# Being me and being us - adolescents’ experiences of treatment for eating disorders

**DOI:** 10.1186/s40337-015-0051-5

**Published:** 2015-03-25

**Authors:** Katarina Lindstedt, Kerstin Neander, Lars Kjellin, Sanna Aila Gustafsson

**Affiliations:** Psychiatric Research Centre, Faculty of Medicine and Health, Örebro University, SE-701 82 Örebro, Sweden

**Keywords:** Eating disorders, Treatment, Adolescents, Family involvement, Patients’ perspective, Qualitative research

## Abstract

**Background:**

This qualitative study addresses adolescents’ perception of treatment for eating disorders. The importance of involving parents in treatment of young people with eating disorders, especially young people with Anorexia Nervosa, is emphasized in a number of studies. Even so, this form of treatment does not work for everybody, not even within a limited diagnostic group. Previous research has revealed that many young people are not entirely satisfied with their treatment. However, there is a lack of knowledge concerning the perspectives of adolescents in outpatient treatment, whose treatment often involves family. The aim of the present study was to investigate how young people with experience from adolescent outpatient treatment for eating disorders, involving family-based and individual based interventions, perceive their time in treatment.

**Methods:**

This study was conducted using a hermeneutic phenomenological approach. Fifteen participants were recruited in collaboration with four specialized eating disorder units and interviewed with the purpose to gather narratives.

**Results:**

The analysis revealed that the adolescents sometimes felt more or less forced into treatment, and strong ambivalent feelings about if and how to participate in treatment permeated the adolescents’ narratives. The common factors which emerged in the narratives were assembled under the two major themes: *Having to involve family in treatment - in one way or another* and *Making progress in treatment - a matter of trust.*

**Conclusions:**

It is of great importance to involve family in treatment in order to understand the problems of the adolescents in their context and be able to take advantage of the resource that parents constitute. However, in certain situations, it is necessary to prioritise individual treatment interventions so that instead of sorting out difficult family situations the therapist focuses on enhancing the young people’s resilience, thus enabling them to tackle problematic situations in life.

## Background

This study addresses adolescents’ perception of treatment for eating disorders. Eating disorders often start in the later teenage years and affect mainly girls and young women [1,2], however increasingly boys and young men as well [1,3]. It may affect young people during key stages in life [4,5] and early treatment interventions have proved to be important for a favourable prognosis [6]. The importance of involving parents in treatment, primarily treatment of adolescents with Anorexia Nervosa, is emphasized in a number of studies, e.g. [7-10], as well as in the NICE treatment guidelines [11]. However Fairburn [12], Gardner & Wilkinson [13], Gowers et al. [14] and Strober [15] among others argue that family-based treatment does not have the strong empirical support as proposed from various quarters. It has for instance been suggested that in family-based therapy eating and weight are being given too much emphasis and that patients improve their eating habits during treatment in order to please family and therapists. The symptoms may be reduced in that way, but there is a risk that cognitive and affective symptoms persist [16].

Previous research has revealed that many young people are not entirely satisfied with the treatment received and the contact experienced during treatment [17-20]. Even people who experienced a positive outcome of treatment, as defined by clinically established criteria describe that they have not been understood or that the therapist could not help them [17,18]. Dissatisfaction with treatment has for instance been linked to treatment delay, to the fact that treatment interventions have not had the desired outcome, and to a premature cessation of treatment [17,21]. According to Federici & Kaplan [22] negative thoughts and feelings of depression or anxiety often remain in people who were dissatisfied with the on-going treatment, something which reduces their ability to maintain normal weight after completion of treatment and increases the risk of relapse. Against this background it is important to incorporate different perspectives when investigating which treatment factors can possibly result in a good or poor outcome. In addition to dealing with outcome measures as reported by therapists, which today form the basis for most of what we know in this area [21,23,24] it is essential also to focus on former patients’ perspective of “what works”.

Studies exploring which aspects of treatment for eating disorders that are considered by former patients to be helpful have identified factors as participation and control in treatment [25,26], social support from friends and family during the treatment process [4,17,19,22,25,27,28], specialized treatment conducted by therapists with extensive expertise [19,26-28], interventions focusing on cognitive symptoms [4,26,28] and individual therapy in combination with family-based therapy [18,19]. However, there is a lack of knowledge concerning the perspectives of adolescents in outpatient treatment, whose treatment often involves family and is made up of different methods and interventions integrated with each other [29,30].

The aim of the present qualitative study was to investigate how young people with experience from adolescent outpatient treatment for eating disorders, involving family-based and individual based interventions, perceive their time in treatment.

## Methods

The study was conducted using a hermeneutic phenomenological approach influenced by Max van Manen [31]. The phenomenological aspect involves an attempt to understand how people give meaning to their experiences of significant events. The interpretive hermeneutic aspect implies putting these narratives in their context, trying to make them understandable from a social and psychological perspective [32]. An important argument for this approach is that it takes into account the complexity of the lifeworld, which means one can speak about multiple and different human lifeworlds that belong to different human existences and realities [31]. Van Manen’s method allowed us to gather narratives, which is proposed to be a good way to really capture the informants’ experiences and thoughts about a phenomenon and get hold of the unexpected [33]. The themes and subthemes that emerge from the analysis process are supposed to illustrate essential information about the data in relation to the research question, and represent a response pattern or meaning within the data set [34].

Among the three authors taking part in the analysis one (KL) has experience from working with young people, one (KN) from family interventions and one (SAG) from the field of eating disorder treatment, including adolescents, families and adult patients.

### Participants

The fifteen informants were aged 13 to 18 at the outset of treatment. Six of the participants were treated for Anorexia Nervosa and nine of them for Eating disorder not otherwise specified (EDNOS) with a restrictive symptomatology, according to DSM–IV–TR [35]. Most of them were in treatment for one to two years. The numbers of therapy sessions given within the framework of their treatment were stated in the intervals 5–10, 11–30, 30–50 or more than 50. Most of the participants had 11–30 sessions; one of them had 5–10 and two of them more than 50. Three of the participants had only family-based therapy sessions (defined as treatment sessions when at least one parent was involved) whereas the treatment for others comprised a blend of family-based therapy sessions and individual sessions. For nine of them the emphasis was on individual therapy sessions; two of them had an equal amount of individual and family-based therapy sessions whereas one of them had more family-based therapy sessions than individual ones. Four of the participants were treated in inpatient care during a certain period.

### Data collection

Participants were recruited in collaboration with four specialized eating disorder units in Sweden, selected from an age group accepted for treatment (13–19 years) and on the basis of the treatment program offered (outpatient family-based and individual treatment). In order to be included, the participants should have completed treatment at one of these units, should have been 13-19 years old during treatment and should not have met criteria for any eating disorder diagnosis at the end of treatment. Clinicians at the four units asked 20 women and four men who met the inclusion criteria if they wanted to be contacted by KL for further information concerning the study, of which 16 women and three males approved. Four of them, two women and two men, declined to participate at a later stage. Participants, who accepted to be contacted by KL, first got a letter describing the project and the conditions for participating. The letter was followed by a phone call in order to answer any questions and go through practical details. Before the interview a written consent was signed by each participant, and the interviews were then conducted by KL. Most the interviews were conducted approximately one to three years after completion of treatment. However, one of the interviews was a trial interview undertaken with a woman shortly after completion of her treatment.

The informants were asked to talk openly around the question “Can you tell me about your time in treatment?” and an interview guide, developed for this purpose, was used when needed to bring the story forward. It contained questions like “Which roles in treatment did your parents take on?” and “How would you describe the relationship to your therapist?”. The interviews lasted between 45 and 90 minutes and were carried out at meeting-places chosen by the participants, such as a library or their homes. The study was approved by the Research Ethics Committee of Uppsala (Dnr 2011/478).

### Data analysis

The interviews were audio recorded and transcribed verbatim. Participants who wanted to read their interviews in a written form had copies sent to them and were given the opportunity to add or change things. The analysis was then conducted in different stages in order to find themes that created meaning. The first stage of the thematic analysis, the *holistic* or *sententious* approach, involved several naive readings of the transcription that were carried out independently by the authors. They wrote down what they perceived the adolescents’ narratives primarily dealt with, e.g. *Camilla needed to reach the bottom before she could begin to recover* or *Bim’s friends provided valuable support during treatment.* In joint discussions after the first reading the authors discussed and formulated tentative themes in the text as a whole, e.g. *Important persons in the foreground/background* or *The dedicated school staff.* Systematic interpretation took place during the second stage, the *selective* or *highlighting* approach, using QSR International’s NVivo 10 qualitative data analysis software [36]. At this stage the authors looked for short narratives that stood out and reflections or phrases that seemed particularly significant, e.g. “Dad was the one who had to stand up for everything and be the strong one” or “The therapists always wanted to see me happy, with a smile”. The contents of these elements were analysed in close collaboration between the authors, where upon subthemes of the text emerged. The relationships between the subthemes were then analysed, which led to the formation of two major themes. The formulations of the themes at different levels were then tested against the totality, in accordance with the hermeneutic circle, and were rejected (e.g. *The dedicated school staff*), retained and reformulated in a process of writing and rewriting. This process continued until the subthemes and the major themes together constituted a totality which according to the authors captured the meaning of the text. In the final *detailed* or *line-by-line* approach, the themes were again tested against each sentence or paragraph.

### Results

The adolescents felt more or less forced into treatment, and strong ambivalent feelings about whether and how to participate in treatment permeated the adolescents’ narratives. What is common in the narratives has been assembled under *two major themes* and *three subthemes within each major theme* (see Table 1).

**Table 1:** **Major themes and subthemes**

**Subthemes**	**Major themes**
Crossing the border to treatment - pushed forward by parents	Having to involve family in treatment - in one way or another
The degree of family involvement - up to whom?	
Being the focus of attention - at someone else’s expense?	
Letting someone else take over the reins – temporarily	Making progress in treatment - a matter of trust
Finding someone right for you	
Coming to an end - it’s all about timing	

Themes and subthemes are described below and are illustrated with a selection of narratives and quotations that further demonstrate the relations between the data and the themes. What the informant says is reproduced in *italics* whereas the interviewer’s comments and questions are written in ***bold italics***. Square brackets around an ellipsis […] show that parts of the text considered extraneous for the context have been removed, whereas three dots in the text … indicate a short pause in the story. When necessary for understanding, clarifications have been provided in brackets. The names used for the informants are fictitious.

### Having to involve family in treatment – in one way or another

As minors the adolescents were obliged to involve their family in treatment and they describe how they strived towards recovery both as individuals and on the basis of their social affiliation as family members.

#### Crossing the border to treatment - pushed forward by parents

The young people’s descriptions of the first encounter with the therapist touch upon strong and often ambivalent feelings such as shame, relief, frustration and exhaustion. In most cases the parents took the initiative for treatment which meant that many adolescents felt angry and showed strong resistance to attending.

*I was blazing mad because I didn’t realize the point of going there. But I thought that I have to go there to somehow show them that there’s nothing wrong, that I don’t need to be there. But they disagreed.* (Iris, 19 years)

The narratives deal mainly with the young people’s own feelings and how the situation affected them. The adolescents do not speak much about what the therapist was like at the beginning of treatment and when they do so, they do it from their perspective of how the therapist’s behaviour made them feel.

*I thought it was very, very hard. First of all because there were two unknown people I had never met before, and then because both my parents had to be there. I had vomited too… and to sit and talk about that when my mum and dad were there listening and everybody looked at me… […] In fact, I felt like some kind of UFO.* (Ofelia, 20 years)

Most of the young people denied their problems and did not want to leave their often well-established routines for something unknown. Many of them did however know somewhere deep inside that they could not go on living the way they did and that they needed help.

*It was as if there were two different sides within me, one saying “Oh God, this is great, I’m going to get help now, I’ll get out of this shit”, but at the same time it was also like this, “No, now they’re going to destroy what you have achieved, and you who have come this far” […] “Do you really want that?” This kept going on; it was like a battleground all the time.* (Bim, 20 years)

#### The degree of family involvement – up to whom?

All adolescents in the study had either both their parents or one of them present at the first meeting. Most of the youngsters were very introverted at the first meeting and their parents often had to speak for them. However the degree of involvement in the therapy later on as well as the level of commitment and support from home varied. Several of the young people had a complicated family situation which affected the role the parents were apportioned or took on during treatment.

*My parents have like alcohol problems too, so I think that is why I was given responsibility. Why I had to look after myself from an early stage. […] We attended some kind of … family thing, but I preferred going on my own. […]****The fact that your parents had problems, was that something you tried to address in any way?****No, the fact is I never told anyone at the eating disorder unit. […] I was so ashamed and I felt I was so dead scared of what might happen if they learnt about it. Because you love your parents, no matter what.* (Diana, 18 years)

In retrospect, most adolescents think that the parents’ participation had a positive effect on the result of the treatment.

*It was so important that my parents were there too, […] because then they could spur me on at home too. […] They* (the therapists) *asked my parents what they felt and what they thought and what they saw […] and in that way we came closer to each other, I think. […] We could speak out and shout at each other, you could be angry and all that, […] and then, they didn’t give up when we came home. […] At the time, I thought it was annoying; it is only now, afterwards, that I see that it was important and that it helped me a lot.* (Emma, 21 years)

However the individual sessions were also of great importance and some of the adolescents thought that family sessions were unneeded. Iris explains why she did not want her parents to take part in the treatment:

*You see, I didn’t trust my parents […] and then it would just have been so strange to have my mum sitting there. Then I would not have dared to be honest in the same way.* (Iris, 19 years)

Those adolescents who never had any individual sessions wish afterwards that they had had such sessions and they think they would have dared to bring up things they did not dare to bring up in the presence of their parents. In the narratives there are also examples showing that an active role in treatment by parents is not always favourable, for example when the parents find themselves in a serious conflict. Camilla, 20 years, describes this in the following way: *With mum and dad it was like this – they threw dirt at each other and I sat there feeling bad. […] When two grown-ups start yelling and arguing, it is of course difficult.*

#### Being the focus of attention – at someone else’s expense?

In retrospect many of the adolescents wish that their siblings had been more involved in the treatment, and when looking back they realize that they occupied a lot of space in the family during the treatment period. Some of them have a bad conscience about the fact that together with their parents they created a world to which their siblings had no access, and they reflect on how marginalised their siblings must have felt with their concerns and worries.

*My sister thought that this was a very tough period when she saw that I was sick. Once she came to embrace me, and then she said “I love you, you are only skin and bone, it is not nice to hug you anymore”. It was so painful to hear that.* (Bim, 20 years)

Several of the adolescents also mention that the fact that they wanted to control much at home – such as decide what would be served for dinner and how the food should be presented – led to many conflicts, and that the hardest thing was to see how the siblings suffered. They describe how the siblings were often eclipsed or chose to withdraw. Helene tells about her brother:

*He kept in the background. He kind of witnessed the mess, or what to call it, from afar. […] He saw how I was feeling, and he was around when I became anxious, so he turned more quiet and observant, one could say. We didn’t have great contact at that time; […] we kind of drifted apart. The disease became like a barrier between us. […] I am incredibly indebted to him […] Even though I could not help it if I was sick; he had to suffer for it too.* (Helene, 19 years)

The work on getting better meant changed family relationships, for better or for worse.

*And finally we came to a point, for there is much fighting and quarrelling when it comes to food and eating and such things, at which stage my parents expressed the feeling “Now you had better get a grip on this situation or else we will separate”. […] Then I felt like “Oh, I’m wrecking my family”. […] One positive thing about all this is that the disease has been good because it has brought us all closer to each other as a family, we have much better communication. […] But my brother was so hurt by all this so he […] hasn’t spoken to me since then. […] Maybe, when he has worked through this, he will be able to let me in again. Time heals, that’s my hope.* (Nelly, 21 years)

### Making progress in treatment – a matter of trust

The adolescents’ narratives about relationships with therapists highlight confidence and trust as a prerequisite for a successful treatment.

#### Letting someone else take over the reins – temporarily

Going through treatment often means that for a certain period one has to entrust others to make important decisions and determine how to proceed, until one feels better and can control things oneself more effectively. Several of the adolescents played a rather passive role in treatment, a role which was more or less self-selected. For some it felt good to hand over to the therapist, for instance because they were unable to control everything when they were seriously ill, and for them it was also important to gradually regain control.

*Right at the beginning, when I was so seriously ill, I couldn’t actually decide for myself. […] If I made a decision, there was someone there inside who punished me. It was nice at first* (to hand over)*, but later on, when I started getting well, I wanted to control things myself a little bit more.* (Linda, 20 years)

There were also those who wanted but did not dare to demand a participatory role, but the great majority just describe for instance the contents or the pace of the treatment without reflecting on what possibilities they had themselves to influence the process. Some of the adolescents find that decisions were made in treatment about courses of action which they did not understand. In retrospect they wish they had been given more information and that the therapists had been more open to alternative solutions.

*I didn’t see the point of any of the suggestions they came up with, […] they sounded like taken from a textbook, like “This is how to do it, this works”. […] It felt like… blinkers on, like “This is the way to do it, there is no other way”.* (Iris, 19 years)

For others the treatment was designed as far as possible according to their own wishes, which strengthened their sense of ownership and responsibility.

*Later on I was allowed to decide to a large extent what I wanted to talk about; […] this made me feel secure. […] “How would you like it to be? Would you like to do full days or would you prefer to come once a week?” […] Now I could decide things to a certain extent, it was more kind of my own responsibility, […] “Yes, we help you, we provide the tools, but you have to carry this out yourself and do the job”.* (Nelly, 21 years)

#### Finding someone right for you

Most of the adolescents mention at least one professional who became an important support whilst in treatment, and the relationship to that person is often described as a complement to other significant relationships in the young person’s life.

*I talked a lot with my best friend too, and then I felt that it was nice to have someone who was close to me. […] But on the other hand she* (the therapist) *at the eating disorder unit was also a great support. […] Where my pal provided friendship support, she had something else to offer me.* (Bim, 20 years)

The relationships in treatment became helpful when the young people met someone they felt was just the right person for them; someone who saw the human being behind the disease and not whisked away their feelings. As Helene, 19 years, describes it, *“One identifies oneself so much with the disease and one appreciates when other people do not do the same thing”.* At the same time other informants stress the importance of experience; they want the therapist to have been actively engaged in treatment work for a long time, or even to have suffered from eating disorders, thus knowing what the disease is all about. Iris tells about a psychologist she met after the treatment at the eating disorder unit had come to an end:

*He himself had had anorexia, so I really had a feeling […] that I was understood in quite another way. […] He could refer to himself, saying “This is what happened to me and I didn’t realize how stupid it was, but it is possible to get out of it”. […] Then I felt at once that something happened, I started to think in a new way. […] It is impossible to understand such a situation unless one has experienced it oneself.* (Iris, 19 years)

The young people highlight the trust and confidence that emerged from a dialogue with the therapist; a trust which deepened with time and led to the young person finding the courage to open up without being afraid of rejection or raised eyebrows.

*In the beginning, like the first two months, I was snappy and curt and I just said “No, I’m not going there”. At that time I hardly answered when they talked to me. But as time went on, and the more I started to trust her […] our talks became deeper. […] I think an important part of the turnaround was when I invited her into my feelings. […] I started to trust her.* (Frida, 17 years)

Nelly talks about her therapist:

*She really cares, really, with her heart, and she is passionate about this. […] One never felt odd […] she was always very open. […] You arrive there, she hugs you and invites you in. She doesn’t distance herself saying something like “Well, I sit here on the other side of the table and I’m a little bit more important than you”. It is almost like she is a friend, she somehow takes care of you, and she is very good at focusing on what is good. You feel you can laugh together but at the same time she has a lot of experience, good ideas […] and she took me seriously.* (Nelly, 21 years)

In retrospect, many of the young people also value that they met resistance; that the therapist dared to challenge them, not accepting evasive answers or excuses.

*At the time I found it awful, but now I think it was great […] that they really dared to push me. […] In the beginning […] I didn’t like her* (the therapist)*, for she really went straight to the point wanting to mess up things for me. But then, when I kind of understood that she did that to help me, I liked her. […] She could tell me exactly what I felt, […] so then I had to be honest. […] It was like being completely cornered.* (Emma, 21 years)

Some of the young people never found anyone with whom they could open up and work constructively. Some describe how they felt neglected in the treatment process, as Nelly, 21 years, expresses it: *“One felt always guarded but never seen”.* Some of them did not feel they could trust the therapist to do the right thing.

*They knew that my particular problem was the exercise, and still they gave me one hour’s walk every day. […] I think they made a mistake there. […] I was having anxiety if I didn’t train, if I had eaten something, but that was just what I had to go through. I needed to have that anxiety, feel really crappy to get over it. The more one is having anxiety, and the more one can put up a fight against it, the better it is, but they did just the opposite.* (Bim, 20 years)

#### Coming to an end – it’s all about timing

Thoughts around coming to an end deal mainly with timing, the importance of ending treatment at the right moment, based on the perspective of both the young person and the therapist. However, they are also about goals. The therapist’s role at the ending of treatment is clearly evident, in contrast to her/his role at the beginning of treatment. Frida describes her therapist’s approach:

*She followed her intuition in the sense that she waited until I was ready, when she saw that I was well again and when I had gained sufficient weight, according to her. But she never said to me that in so and so many months you will be discharged. […] It was more like we sat there talking, “How are you feeling now? Do you feel you are ready to be discharged?”* (Frida, 17 years)

Some of the informants mention how the therapists set up goals relating to weight as a benchmark for ending treatment, something that the young people did not feel comfortable with and which they question retrospectively. In their narratives they speak very little about weight in relation to coming to an end of treatment, but more about the importance of feeling strong and having improved their relationship to food.

*When I reached normal weight we met increasingly infrequently, and then the treatment was phased out. But it felt a bit like a slap in the face that “OK, now you have reached normal weight – so now you are well again”. […] The simple fact that I had put on weight meant that everything was fine, and that was all there was to it. I felt like … “I’m not prepared to walk out weighing like this, if you leave me, I will start losing weight again”. […] There is still a sense of apprehension that you may not be completely recovered and that’s a fact.* (Linda, 20 years)

Many of the adolescents had follow-up contacts with their therapists after treatment had ended, others were offered follow-up calls but did not feel they needed them and yet others never got this offer but wish they had had the opportunity. Nelly relates how her therapist was there for her:

*I had the feeling she was really present when I had to take my first faltering steps […]. It was really nice to feel her support, because that is one of the big things when you are not well, you feel you are almost happy about it because you get the support. And then, when you are well again, all that disappears.* (Nelly, 21 years)

Many of the young people felt insecure after ending treatment – even though they had felt prepared for it – and were helped by the fact that when necessary they could take up the contact again. As Helene, 19 years, describes her therapist: “*We are still in contact. She is my crutch now, quite simply”*.

## Discussion

Adolescents in treatment for an eating disorder are struggling with the balance between independence and dependence, according to the participants in this study. As minors they were obliged in one way or another to *involve their family in treatment* and they describe what this implied for them and how family relationships of various kinds came to affect treatment. Although the family clearly provided an important context, the adolescents were in a process of emancipation. In addition, even though their parents took the initiative to treatment, the young people were expected to do most of the work as individuals and recover “for their own sake”. Cooperation with the therapist was described as meaningful in this quest for balance and the adolescents state that *making progress in treatment is a matter of trust.*

### Family involvement

The narratives illustrate the parents’ significant role in the initial stage of the treatment, but the benefit of parents’ involvement in treatment later on depended on the prevailing family climate. Many of the adolescents found themselves in a difficult family situation and only a few of them experienced family-based therapy sessions as helpful. The informants speak about how either their parents or themselves withheld issues they actually wanted to talk about. The reason for involving parents in treatment is to help the therapist understand the development of the disorder and identify supporting factors [37-39], and Christie, Warkins, and Lask [40] emphasize the importance of parents keeping their own problems outside during family-based therapy sessions. There is a potential for conflict between the ambition to keep parents’ own problems outside and the ambition to understand the problems of the adolescents in their context. It can be problematic for young people in therapy to speak about certain things with their parents, especially if they feel that the prevailing family situation is part of the problem [41], and the ambition to keep the parents’ own problems outside may become “the elephant in the room” that no one mentions. Since these adolescents are also in the process of emancipation it may also be that they need to be able to talk about certain things without parental involvement. Most of the adolescents were of the opinion that the treatment should include individual therapy sessions in combination with family-based therapy sessions, something which has also been suggested in previous studies [18,19]. The ones who experienced family-based therapy sessions as productive felt that on these occasions the parents learnt how to manage eating related issues between treatment sessions. But not even in those cases were parental involvement totally uncomplicated. Suddenly parents were called upon to be co-therapists and ensure that what was implemented in treatment was followed at home, and for some of the adolescents this meant that their parents started confronting certain behaviours which they had previously accepted.

An eating disorder affects the whole family in several ways. When the disorder is brought into such a prominent focus and parents during treatment devote most of their time to supervise the young person, it can wear down family relationships to a great extent. Many of the adolescents experience in retrospect that their siblings were disregarded or chose to withdraw, and several of them lay the blame on themselves for that. In our study there are examples of how such cracks in the family can become permanent, but there are also examples of how family ties became stronger. This point to the importance of addressing the situation for the whole family in therapy [42,43].

### Therapeutic alliance and goals

In most cases it was the parents who took the initiative for treatment and even though some of the young people had reached a point where they realized that they needed help, most of them were reluctant and afraid of what the treatment might entail. Persons with eating disorders, particularly Anorexia Nervosa are often ambivalent to starting treatment, e.g. [20,44,45], and experience difficulties in creating a strong alliance with their therapist [44,46]. The adolescents describe that it took several sessions before they dared to open up to their therapist and could take in the situation without being obstructed by all their emotions, and that the directions for further treatment could be outlined only later on. The notion of “emerging mutual trust” has been used in a previous study to capture the fact that trust might not come about immediately in a therapeutic relationship [47]. However, the way the therapist acted, spoke and treated the adolescents at the first encounter was of great importance. Even if, at the time, the adolescents could not really take it, for some of them partly due to starvation, they experience afterwards that the therapist’s attitude laid the foundation for the continued relationship. It has previously been found essential that in spite of resistance, the therapist issues an invitation to cooperate, and that it is done over and over again until the young person realizes that the therapist is there to help [48]. Our study highlights several examples of how such insistent attempts can be helpful and that there is otherwise a risk that the young person leaves the treatment with a feeling of not having been seen or understood. A strong alliance between therapist and patient is of great importance for the treatment outcome [46,49-52]. This requires trust; the therapist should weave in the patient’s views on treatment and the two parties should have an agreement about common goals and the design of the treatment [53,54]. It has been shown previously that the therapist’s qualities can affect the alliance [50] and the informants in our study emphasize the importance of meeting a therapist who “feels right”. Qualities in the therapist described as important may appear contradictory; to be “kind” and at the same time “firm”, “professional” but at the same time “human” and so forth. According to the adolescents, it is also important that the therapist has extensive experience but at the same time is able to put aside all prior understanding and consider each patient’s unique experiences and conditions. Several of these qualities have earlier proved to be important from the eating disorder patient’s perspective [55].

Not one of the young people mentions anything about what method the treatment was based on. This may possibly indicate that different methods were integrated with each other and with other treatment interventions, something which according to e.g. Clinton [29] and Norcross and Wampold [30] is a common approach in everyday clinical practice, or that the treatment was based on a so-called contextual model which primarily is not built on a standardized method but rather on the patients’ contexts and how they experience the process [56-58]. A possible conclusion is that the method itself was irrelevant for the young person as long as the therapist worked in order to meet her/his needs. For example, Wilhelmsson Göstas and her colleagues made a similar finding in their study pointing to a perceived inseparability between the method and the therapist [59].

It is important in treatment to jointly clarify the expectations and what the treatment should lead to [56-58], that is to create a shared view of the situation. Otherwise, there is a risk that the treatment is terminated although the patient believes there are problems left to work with, or that a patient leaves the treatment prematurely because the process is not progressing [58]. In this study, there are examples of therapists who focused on weight gain as a goal for treatment and equated increase in weight with improved mental health, something which did not correspond to the adolescents’ own experience. Some of them felt unprepared for termination of treatment and are afterwards disappointed that the therapist was not more interested in their thoughts and feelings.

### Strengths and limitations

The interviews were carried out by a person outside the context of treatment, which is a possible strength since it might have made it easier for the informants to speak openly about their experiences. Also, since the informants were asked to speak freely about their experiences, the influence of the authors’ pre-understanding was reduced. The fact that for most of the informants some time had passed since the end of treatment can be seen both as a strength and as a limitation. On one hand the young people had gained perspective on their period of illness and acquired a clearer picture of it, on the other hand their information may have been marked by memory bias. The fact that we do not have any information regarding the type of family therapy used at the units is a limitation. The sample size can be considered adequate to meet the purpose of the analytical method, but the homogeneity of the sample is a weakness. One single participant was male, which according to recent studies does not fully correspond to the factual gender ratio in the field [3], and none of the participants had a foreign background. Since the results are limited to such a small group they do not enable generalizations, although they may be of importance for people who can apply them on their own context.

## Conclusion

In their narratives, not one of the informants mentioned which method or technique the treatment was based on. Instead, they enhance the importance of the therapists’ personal attributes. The adolescents in this study describe that a tough but important challenge for the therapist in treatment of adolescents with eating disorders is to come in and momentarily “take over” the decision making, and at the same time make the patient participate in parts which she/he can take responsibility for. Also, this degree of involvement needs to increase gradually in line with recovery. The task becomes particularly complex when the therapist also has to relate to parental involvement, but might at the same time be even more important then, in order not to let the parents’ needs overshadow those of the adolescents. In certain situations, it is necessary to prioritise individual treatment interventions so that instead of sorting out difficult family situations the therapist focuses on enhancing the young persons’ resilience, thus enabling them to tackle problematic situations in life. The young people need honesty, also when it is tough and uncomfortable, but this presupposes a sustainable relationship based on mutual trust. To be able to create such a relationship with a patient the therapist needs to see the patient as a competent person and validate what the patient describes.
